# Determination of exercise intensity domains during upright versus supine cycling: a methodological study

**DOI:** 10.7717/peerj.13199

**Published:** 2022-04-13

**Authors:** Damir Zubac, Vladimir Ivančev, Vincent Martin, Antonio Dello Iacono, Cécil J.W. Meulenberg, Adam C. McDonnell

**Affiliations:** 1 Kinesiology, University of Split, Split, Croatia; 2 Institute for Kinesiology Research, Science and Research Center Koper, Koper, Slovenia; 3 AME2P, Université d’Auvergne (Clermont-Ferrand I), Clermont-Ferrand, France; 4 Institut Universitaire de France, Paris, France; 5 Institute for Clinical Exercise and Health Science, School of Health and Life Sciences, University of the West of Scotland, Hamilton, United Kingdom; 6 Department of Automation, Biocybernetics and Robotics, Jožef Stefan Institute, Ljubljana, Slovenia

**Keywords:** Constant load exercise, Skeletal muscle oxygenation, Critical power, Metabolic flexibility

## Abstract

**Background:**

There is a growing interest among the research community and clinical practitioners to investigate cardiopulmonary exercise test (CPET) procedures and protocols utilized in supine cycling.

**Materials and Methods:**

The current study investigated the effects of posture on indicators of exercise intensity including gas exchange threshold (GET), respiratory compensation point (RCP), and the rate of peak oxygen uptake (V̇O_2_ peak), as well as the role of V̇O_2_ mean response time (MRT) in determining exercise intensity domains in nineteen healthy men (age: 22 ± 3 years). Two moderate-intensity step-transitions from 20 to 100 Watt (W) were completed, followed by a maximal CPET. After completing the ramp test, participants performed a constant-load at 90% of their attained peak power output (PPO).

**Results:**

No differences were observed in the V̇O_2_ MRT between the two positions, although the phase II-time constant (τV̇O_2p_) was 7 s slower in supine position compared to upright (*p* = 0.001). The rate of O_2_ uptake in the supine position at GET and RCP were lower compared to the upright position (208 ± 200 mL·min^−1^ (*p* = 0.007) and 265 ± 235 mL·min^−1^ (*p* = 0.012) respectively). Besides, V̇O_2_ peak was significantly decreased (by 6%, *p* = 0.002) during supine position. These findings were confirmed by the wide limits of agreement between the measures of V̇O_2_ in different postures (V̇O_2_ peak: −341 to 859; constant-load test: −528 to 783; GET: −375 to 789; RCP: −520 to 1021 all in mL·min^−1^).

**Conclusion:**

Since an accurate identification of an appropriate power output (PO) from a single-visit CPET remains a matter of debate, especially for supine cycling, we propose that moderate-intensity step-transitions preceding a ramp CPET could be a viable addition to ensure appropriate exercise-intensity domain determination, in particular upon GET-based prescription.

## Introduction

For almost a century, cardiopulmonary exercise testing (CPET) has been the gold standard of integrative physiologic assessment (Hill & Lupton, 1923), and is widely implemented as the diagnostic tool in both clinical and research settings to provide functional insights regarding general health and exercise tolerance (Kaminsky et al., 2017; Guazzi et al., 2017; Wasserman et al., 2020). However, researchers are still investigating the nuances of the moderating factors, including biological, physiological, biomechanical, and methodological aspects related to CPET (Meyler, Bottoms & Muniz-Pumares, 2021). The pertinent stages, such as the rate of oxygen uptake (V̇O_2_) at the gas exchange threshold (GET) and the respiratory compensation point (RCP), provide objective outputs for clinicians and exercise physiologists to tailor personalised exercise interventions that can optimize overall cardiorespiratory health and performance while avoiding premature fatigue development. However, this is not a universal finding, especially for V̇O_2_ uptake at RCP during upright exercise (Leo et al., 2017).

For upright cycling, maximal individual capabilities and exercise intensity targets are typically derived from incremental ramp CPET, performed during a single laboratory session (Whipp et al., 1981). Yet, the individual identification of optimal metabolic zones, and an accurate prescription of equivalent targeted constant work-rate exercise from CPET is still challenging. Typically, for a given cycling power output (PO), V̇O_2_ assessed during a ramp CPET is often either underestimated or overestimated for constant-work rate exercise at that specific PO, due to several factors including high between-participant variability of maximal intensity capabilities (Keir et al., 2018). Thus, the heterogeneity in the response to training and potential methodological pitfalls further complicate an accurate identification of cycling metabolic zones and equivalent PO’s. Determining the PO for a given V̇O_2_ associated with GET–a pivotal indicator whether the exercise intensity- domain was correctly prescribed–is possible *via* V̇O_2_ mean response time (MRT) calculation (Keir et al., 2015; Iannetta, Murias & Keir, 2019). To date, a direct proof of the role of the V̇O_2_ MRT in determining exercise intensity boundaries during supine cycling is missing. Though, the role of the V̇O_2_ MRT calculated from a single-session CPET remains a controversial issue, especially when adjusting the oxygen uptake/work rate at predetermined GET or RCP (Boone & Bourgois, 2017), as these parameters represent important individual physiological limitations to performance that, crucially to note, are usually sustained over time.

There is a growing interest among the research community and clinical practitioners to investigate CPET procedures and protocols utilized in supine cycling (Dillon et al., 2021, Goulding et al., 2021a, 2021b; Steele et al., 2018). The supine cycling model has been increasingly utilized to study age-related changes in cerebral blood flow (Smirl et al., 2016), acute spleen volume changes (Zubac et al., 2021b), cardiovascular adjustments during exercise in athletes (La Gerche et al., 2013), the effects of gravity acceleration (Bonjour et al., 2010) in addition to study skeletal muscle bioenergetics (Goulding et al., 2020). Yet, concepts like GET and RCP determination received less attention during supine exercise, even though supine cycling has been gaining interest as it provides an appropriate alternative for testing certain populations, who were previously thought to be at risk during upright CPET (Mizumi et al., 2018). For example, Smirl et al. (2016) prescribed an exercise intensity domain by determining the % of HR relative to the maximum HR attained during incremental exercise. Crucially, exercise intensity domains were not categorised through gas exchange parameters for the corresponding PO, which has recently been identified as potentially misleading (Jamnick et al., 2020; Zuccarelli et al., 2018; Meyler, Bottoms & Muniz-Pumares, 2021). Although recent studies have identified that V̇O_2_ peak is progressively reduced when moving from an upright position into a supine during cycling (Dillon et al., 2021), there is limited understanding of supine exercise intensity boundaries precisely determined from ramp CPET. For example, the V̇O_2_ MRT during supine cycling was not performed in agreement with Iannetta, Murias & Keir (2019), who recommended that a single-visit ramp CPET to volitional exhaustion should be preceded by a moderate-intensity step-transition protocol to accurately determine the GET.

As such, the purpose of the current study was to examine the effect of posture (*i.e*., supine *vs* upright) on the most often used indicators of exercise intensity prescription: GET, RCP, V̇O_2_ peak, as well as the role of the V̇O_2_ MRT in determining individual exercise intensity domains. We hypothesized that supine exercise would be characterised by: (i) a prolonged V̇O_2_ MRT compared to upright mainly due to sluggish O_2_ transport, and (ii) a slower O_2_ transport, resulting in a significantly reduced rate of oxygen uptake at GET, RCP and at VO_2_ peak compared to upright exercise.

## Materials and Methods

### Participants

The present study followed the principles of the Declaration of Helsinki and was approved by the University of Split, Faculty of Kinesiology Research Ethics Board (approval number: 2181205-02-05-20-020). Twenty-one healthy, active males volunteered to participate in the present study, after being fully informed about the study procedures, the standardized dietary intake prior to all testing procedures, and the potential risks involved, before their written consent was signed. The exclusion criteria were: arterial hypertension (≥140/90 mmHg), a sedentary lifestyle, a history of cardiovascular or peripheral arterial disease, a history of neuromuscular injuries, smoking, dietary supplement consumption (creatine, whey protein and nitric oxide-based supplements) and/or drug medication. Based on these criteria two participants were excluded, leaving 19 heathy active men (age = 23 ± 2, body-mass = 82 ± 7 kg, height = 186 ± 4 cm) enrolled in the present study.

### Study design

All experimental procedures took place under a well-controlled laboratory setting, at approximately the same time of the day (~08:00–12:00 am) on three occasions, including one preliminary medical screening visit and familiarization, followed by two randomly assigned experimental sessions consisting of either an upright or supine position cycling protocol, separated by at least 48-h. The overview of the experimental protocol is depicted in Fig. S1. Participants were instructed to refrain from vigorous exercise, caffeine or alcohol consumption for 24-h and followed a standardized dietary intake prior to all the testing procedures to minimize variability of glycogen stores and glucose oxidation (Colosio et al., 2020).

### Procedures

During the first visit, a medical screening was performed including the collection of a medical history, resting electrocardiograph (ECG), blood pressure (BP) and oxygen saturation (S_p_O_2_: Dash 2000; GE, Milwaukee, WI, USA). Upon receiving medical clearance, participants completed one familiarization session for each experimental protocol on the cycle-ergometer to minimize learning effects, and to avoid O_2_ uptake underestimation throughout the study. More specifically, while supine, the distances between the crankshaft, shoulder and pelvis were carefully recorded for each participant during the familiarization procedures and replicated within the experimental study day.

### Data collection

The experimental protocol for the randomly executed upright and supine cycling conditions was identical. Briefly, upon arrival to the laboratory the participants rested quietly while being instrumented with the metabolic analyser mask (Hans Rudolph, Shawnee, KS, USA) and the heart rate (HR) belt (Garmin HRM-3 SS, Shawnee, KS, USA). The experimental protocol consisted of two consecutive, moderate-intensity step-transitions from 20 to 100 W, followed by a incremental ramp test to exhaustion (Iannetta, Murias & Keir, 2019). Task failure was defined when cadence fell below 75 rpm for more than 10 consecutive seconds despite strong encouragement from the research staff. Upon completion of the maximal effort, participants rested for 20-min in the tested position, and then performed a constant-work rate test at 90% of their previously attained peak power output (PPO) to validate the V̇O_2_ peak attained during the incremental ramp test (in agreement with recent work by Niemeyer, Leithäuser & Beneke, 2020). Lastly, the reason the ramp test was terminated (either due to leg pain or dyspnea) and the subjective rating of perceived exertion (RPE scale, 1–10) (Borg, 1998) were recorded.

### CPET testing protocols

The experimental protocol (Fig. S1**)**, started with baseline measurements of resting V̇O_2_ uptake (5 min), followed by two 6-min step-transitions from 20 W to 100 W, followed by a 3-min reduced intensity phase at 50 W to prepare for the incremental ramp test (20 W**·**min^−1^) to exhaustion. Cycling cadence was set at 75 rpm. Following the cessation of the incremental test, the participants performed a 5-min cool down at a reduced load (40 W) and then rested for 20 min, after which they completed a constant-work rate test at 90% of the PPO reached during the incremental ramp test in both upright and supine positions.

The custom-built supine cycling setup and data collection protocols were strictly standardized, as noted above. To illustrate, in the supine position the centre of rotation of the crankshaft was 33 ± 2 cm above the level of the heart. Also, to reduce movements, strapping (nHance, Barcelona, Spain), was placed around both the pelvis and the shoulders, to secure the participant to the cycle-ergometer. The strapping did not affect pulmonary function, but did limit excessive torso movements and minimized the separation of the saddle and buttocks during more strenuous pedaling towards V̇O_2_ peak.

### Measurements

Pulmonary gas exchange was analyzed *via* a cardiopulmonary exercise testing unit (K5; Cosmed, Rome, Italy), that was synchronized with the cycle-ergometer (900; Ergoline, Hamburg, Germany). Calibration of the K5 metabolic unit was performed in agreement with the manufacturer’s guidelines. All gas exchange/ventilation parameters (V̇O_2_, V̇CO_2_, V̇_E_) were evaluated on a breath-by-breath basis.

### Data processing and analysis

#### Moderate-intensity step transitions

Breath-by-breath V̇O_2_ data were visually inspected for outliers (>3 SDs from the local mean on the basis of six consecutive breaths) and if present, were removed. Next, raw data were allotted to 1-second bins, and then averaged every 5-seconds for each participant individually, with time-zero representing the onset of the exercise. The oxygen uptake kinetics (baseline, τV̇O_2p_, time delay, V̇O_2p_ kinetics amplitude) were modelled *via* mono-exponential, least-squared regression (Eq. 1), as originally proposed (Whipp et al., 1981) and recently adopted by others (DeLorey, Kowalchuk & Paterson, 2004; Goulding et al., 2020; Goulding et al., 2021a; Zubac et al., 2021a). A representative trace of the O_2_ uptake kinetics is given in Fig. S2. Equation (1) was used to calculate the MRT and avoid methodological drawbacks associated with gas exchange determination (Keir et al., 2015; Iannetta, Murias & Keir, 2019; Zubac et al., 2021a, 2021b). Lastly, oxygen uptake kinetics data were averaged and exported to Excel and computed to yield an individualized response.

(1) $$\rm Y(t)= Y_{BSLN} + Amp [1 - e^{- (t - TD) / \tau}]$$

#### Incremental Ramp test

GET was individually determined *via* the V-slope analysis (Beaver, Wasserman & Whipp, 1986) by two exercise physiologists. The influence of delayed V̇O_2_ was corrected for each participant (*via* MRT) in order to calculate the power output associated with V̇O_2_ at GET (Whipp & Wasserman, 1972; Iannetta, Murias & Keir, 2019). More precisely, the GET was established using the following criteria: a systematic increase in the ventilatory equivalent of the rate of oxygen uptake (V̇E/V̇O_2_) and end-tidal pressure of oxygen without a concomitant increase in the ventilatory equivalent of carbon dioxide production (V̇E/V̇CO_2_). The RCP was determined as the point where both V̇E/V̇O_2_ and V̇E/V̇CO_2_ began to systematically increase_,_ while a parallel decrease in the P_ET_CO_2_ was observed (Whipp, Davis & Wasserman, 1989). V̇O_2_ peak was determined *via* rolling average readings (20 s intervals), sampled during the last minute of the incremental ramp test and confirmed in the same manner in the constant work-load test, while the PPO and HR were defined as data attained at the test(s) cessation.

### Statistics

The SPSS software (20.0; IBM, Champaign, IL, USA) was used for statistical analysis. All data are reported as mean ± SD, and 95% confidence interval. Normality distribution was confirmed using the Shapiro–Wilk test. A paired Student’s *t*-test was used to compare data derived from the metabolic unit during the CPET protocols between upright *vs* supine cycling bouts. Bland–Altman plots were generated to evaluate the agreement between the V̇O_2_ demands at GET, RCP, V̇O_2_ peak and V̇O_2_ peak verification bout during upright *vs* supine cycling exercise (Bland, 2000). The level of statistical significance was accepted at *p* < 0.05.

## Results

Nineteen moderately-trained males (a self-reported average of 11 h of moderate-vigorous exercise per week) who had normal mean resting values for heart rate, arterial blood pressures and oxygen saturation completed all study procedures.

The oxygen uptake kinetics data during moderate intensity step-transitions in both upright and supine position are given in Table 1. Briefly, no differences were observed between the positions for either the amplitude of V̇O_2_ (*p* = 0.114, 95% CI [−0.01 to 0.08]), time delay (*p* = 0.054, 95% CI [−0.05 to 6.21]) or MRT (*p* = 0.364, 95% CI [−9.91 to 3.80]). However, in the supine position baseline oxygen uptake was higher (*p* = 0.001, 95% CI [−0.24 to −0.12]), and the phase II-time constant (V̇O_2p_) was 7 s slower, compared to the upright position (*p* = 0.001, 95% CI [−12.0 to −0.19]). The V̇O_2_ averaged over the last 2 min of these moderate intensity step-transitions was significantly higher in the supine as compared to the upright position (*p* = 0.001, 95% CI [−0.22 to −0.10]).

**Table 1:** V̇O_2_ kinetics during upright and supine cycling.

	Upright	Supine	95% CI lower	95% CI upper	*p*-value
Baseline, L**·**min^−1^	1.04 ± 0.07	1.2 ± 0.11*	−0.24	−0.12	0.001
Amplitude, L**·**min^−1^	0.77 ± 0.06	0.74 ± 0.09	−0.01	0.08	0.114
Time delay, sec	19.5 ± 5.1	16.5 ± 5.9	−0.05	6.21	0.054
Tau (τ), sec	29.9 ± 8.7	36.0 ± 11.0*	−12.0	−0.19	0.044
MRT, sec	49.4 ± 8.6	52.5 ± 11.7	−9.91	3.80	0.364
V̇O_2,_ L**·**min^−1^(last 2 min.)	1.81 ± 0.05	1.98 ± 0.14*	−0.22	−0.10	0.001

**Notes:**

95% CI, confidence interval; V̇O2p, oxygen uptake kinetics; MRT, mean response time. Data are presented as mean ± SD and CI.

* Significantly different from upright.

The differences in the cardiorespiratory response between the body positions at GET and RCP are presented in Table 2. For the GET, no differences were observed between the two positions for V̇_E_, V̇CO_2_, respiratory exchange ratio (RER), and GET P_ET_CO_2_. In the supine position at the GET, V̇O_2_ was significantly lower (*p* = 0.007, 95% CI [63.98–350.19]) than in the upright position, and the attained HR (*p* = 0.022, 95% CI [1.26–14.51]) and PO (*p* = 0.001, 95% CI [20.25–33.89]) were also lower, while GET P_E_TO_2_, *p* = 0.013, 95% CI [−0.67 to −2.77]) was higher. No differences were observed for V̇O_2_ at GET and RCP between the body positions when V̇O_2_ readings were given as a percentage of V̇O_2_ peak. At RCP, no differences were observed between the two positions for V_E_, V̇CO_2_, RER and P_E_TCO_2_. Similar to GET, V̇O_2_ (*p* = 0.012, 95% CI [0.69–5.28]), HR (*p* = 0.001, 95% CI [2.64–13.47]), and power output (*p* = 0.001, 95% CI [22.6–43.3]) attained at RCP were lower in the supine position.

**Table 2:** Cardiorespiratory parameters measured at the gas exchange thresholds and respiratory compensation points during upright and supine cycling.

	Upright	Supine	95% CI lower	95% CI upper	p-value
GET V̇_E_, L**·**min^−1^	63 ± 11	61 ± 8	−2.33	7.06	0.305
GET V̇O_2_, mL**·**min^−1^	2,608 ± 283	2,400 ± 278*	63.98	350.19	0.007
GET V̇CO_2_, mL**·**min^−1^	2,491 ± 322	2,338 ± 374	−20.33	324.96	0.080
GET RER	0.95 ± 0.06	0.97 ± 0.06	−0.05	0.02	0.383
GET V̇O_2,_ mL**·**kg**·**min^−1^	32.1 ± 4.5	29.6 ± 4.8*	0.68	4.22	0.009
GET V̇O_2,_ (%)	67 ± 7	66 ± 6	−1.51	3.96	0.361
GET HR, bpm	152 ± 16	144 ± 14*	1.26	14.51	0.022
GET P_E_TO_2_	100.5 ± 5.2	103.3 ± 3.0*	−0.67	−2.77	0.013
GET P_E_TCO_2_	44.0 ± 3.3	42.8 ± 2.4	−0.433	2.76	0.143
GET PO, W	195 ± 23	167 ± 19*	20.25	33.89	0.001
GET PO, W·kg^−1^	2.4 ± 0.4	2.1 ± 0.3*	0.25	0.41	0.001
RCP V_E_, L**·**min^−1^	91 ± 15	92 ± 18	−10.25	7.84	0.783
RCP V̇O_2_, mL**·**min^−1^	3,332 ± 371	3,081 ± 370*	61.01	440.14	0.012
RCP V̇CO_2_, mL**·**min^−1^	3,562 ± 450	3,391 ± 525	−103.11	444.70	0.207
GET RER	1.07 ± 0.05	1.10 ± 0.09	−0.08	0.01	0.185
RCP V̇O_2,_ mL·kg·min^−1^	41.1 ± 6.4	38.1 ± 6.7*	0.69	5.28	0.014
RCP V̇O_2,_ (%)	86 ± 5	85 ± 7	−2.36	4.12	0.577
RCP HR, bpm	172 ± 16	164 ± 18*	2.64	13.47	0.006
RCP P_E_TO_2_	105.6 ± 4.7	109.1 ± 3.9*	−5.72	−1.27	0.004
RCP P_E_TCO_2_	43.3 ± 4.5	41.2 ± 3.0	−0.01	4.12	0.052
GET PO, W	262 ± 25	229 ± 22*	22.6	43.3	0.001
GET PO, W·kg^−1^	3.2 ± 0.4	2.8 ± 0.4*	0.28	0.52	0.001

**Notes:**

95% CI, confidence interval; GET, gas exchange threshold; RCP, respiratory compensation point; V_E_, pulmonary ventilation; V̇O_2_, rate of oxygen uptake; V̇CO_2_, carbon dioxide production; RER, respiratory exchange ratio; HR, heart rate; P_E_TO_2_, end-tidal oxygen; P_E_TCO_2_, end-tidal carbon dioxide; PO, power output; Data are presented as mean ± SD and 95% CI.

* Significantly different from upright.

Table 3 reports comparisons for the overall cardiorespiratory parameters between the upright and supine cycling protocols. No differences were observed between the positions for V_E_, RER and V̇CO_2_ (all *p* > 0.05). However, peak oxygen uptake (*p* = 0.002, 95% CI [111.5–406.7]) was significantly decreased in the supine position, even when corrected for body weight (by ~4 mL·kg·min^−1^, *p* = 0.001, 95% CI [1.3–4.7]). Additionally, the participants attained a significantly lower HR (*p* = 0.001, 95% CI [4.1–12.8]) during the supine position, and irrespective of correction for body weight, reached a lower power peak output (PPO, *p* = 0.001, 95% CI [0.36–0.61]) compared to the upright position. The time to exhaustion during the incremental ramp test was reached almost 2 min earlier when supine was compared to upright (*p* = 0.001, 95% CI [1.40–2.45]). Lastly, no differences were observed between body positions for V̇O_2_ peak during the constant-work rate test (*p* = 0.114, 95% CI [33.8–288.6]), despite substantial differences in the PPO (*p* = 0.001, 95% CI [26.1–44.0]).

**Table 3:** Overall cardiorespiratory parameters are presented for upright and supine cycling protocols.

	Upright	Supine	95 % CI lower	95% CI upper	*p*-value
V_E_, L**·**min^−1^	149 ± 26	138 ± 29	−2.47	22.6	0.109
V̇O_2_, peak. mL**·**min^−1^	3,894 ± 412	3,635 ± 394*	111.5	406.7	0.002
V̇CO_2_, mL**·**min^−1^	4,589 ± 501	4,343 ± 605	−10.1	502.6	0.059
V̇O_2_ peak., mL**·**kg**·**min^−1^	47.9 ± 6.6	44.8 ± 6.8*	1.3	4.7	0.001
RER,	1.18 ± 0.06	1.19 ± 0.09	−0.06	0.03	0.541
HR max., bpm	189 ± 10	180 ± 13*	4.1	12.8	0.001
PPO, W·kg^−1^	3.8 ± 0.5	3.3 ± 0.5*	28.5	48.5	0.001
PPO, W	312 ± 30	274 ± 28*	0.36	0.61	0.001
TTE, min	13.0 ± 1.49	11.1 ± 1.40*	1.40	2.45	0.001
vV̇O_2_ peak., mL·min^−1^	3,765 ± 506	3,638 ± 458	−33.8	288.6	0.114
vPPO, W	282 ± 27	247 ± 25*	26.1	44.0	0.001

**Notes:**

95% CI, confidence interval; V̇E, pulmonary ventilation; V̇O_2_, peak; - peak oxygen uptake; vV̇O_2_ max. - peak oxygen uptake during constant work-rate test; RER, respiratory exchange ratio; HR max. - maximal heart rate; PPO, peak power output; TTE, time to exhaustion; vPPO, peak power output during constant-work rate test. Data are presented as mean ± SD and CI.

* Significantly different from upright.

There was no difference in the RPE data between body positions during CPET test (*p* = 0.368), while exercise termination was predominantly associated with pain in the leg muscles, as compared to dyspnea (9.1 ± 1.2 *vs* 7.6 ± 1.1, *p* = 0.001, in upright), and (9.5 ± 1.0 *vs* 7.6 ± 1.5, *p* = 0.001, in supine).

**Figure 1:**
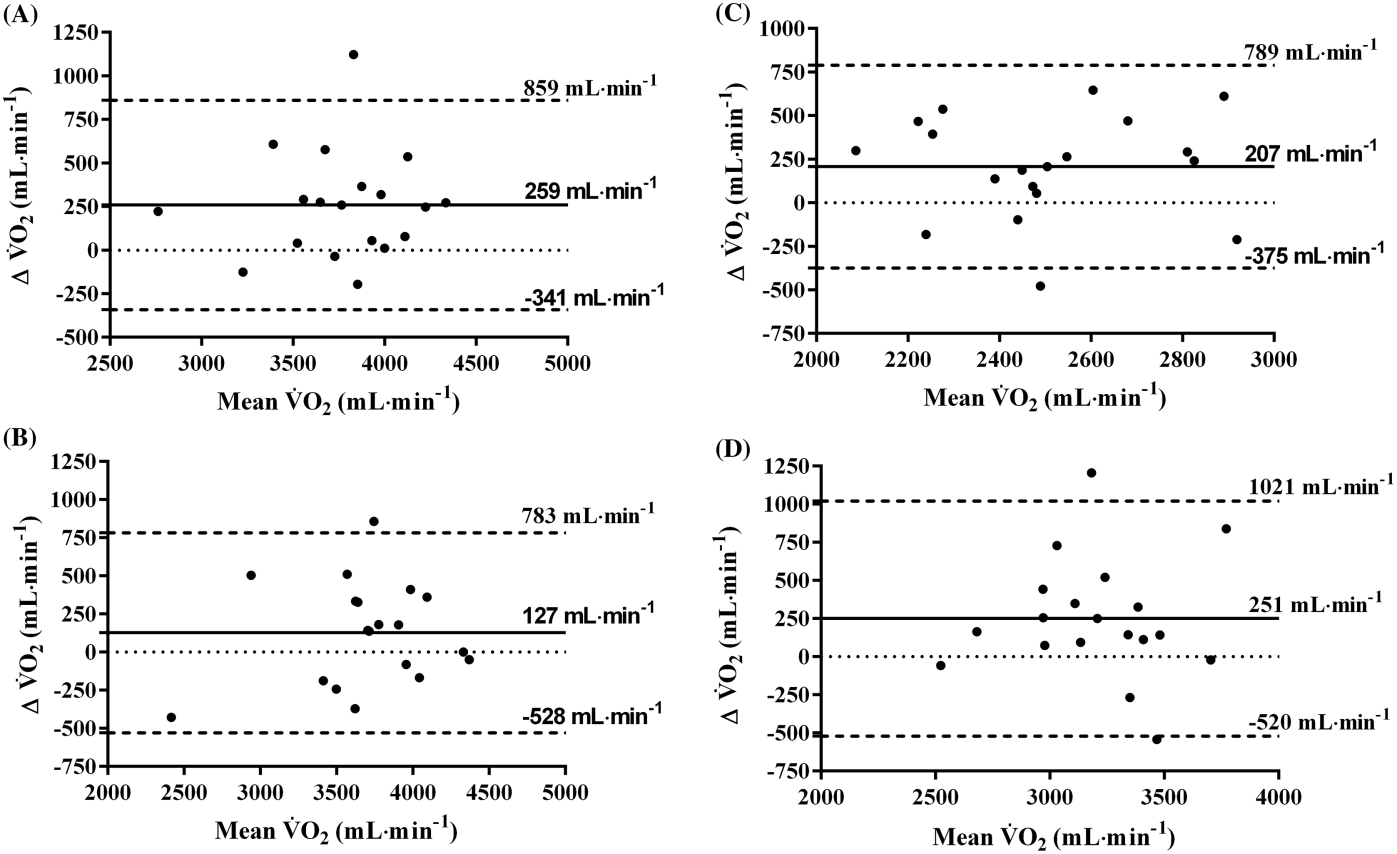
Bland-Altman plots between upright and supine cycling conditions at V̇O_2_ max. (A) V̇O_2_ at verification test (B) V̇O_2_ at gas exchange threshold (C) V̇O_2_ at respiratory compensation point (D).

Figure 1 depicts Bland–Altman plots examining the agreement between V̇O_2_ measured in the upright *vs* supine position as well as the agreements’ deviation from the mean V̇O_2_. For all parameters, the Bland–Altman analysis identified a positive bias in favour of the upright position: the rate of peak oxygen uptake (Fig. 1A: bias = 258 ± 306 ml**·**min^−1^); V̇O_2_ peak. verification bout (Fig. 1B: bias = 127 ± 335 ml**·**min^−1^); gas exchange threshold (GET, Fig. 1C: bias = 207 ± 297 ml**·**min^−1^), and respiratory compensation point (RCP, Fig. 1D: bias = 251 ± 393 ml**·**min^−1^), with generally wide limits of agreement. In addition, for all parameters there was a homogeneous distribution of differences between upright and supine positions across all values of V̇O_2_ peak denoting homoscedasticity (*R* = −0.001, *p* = 0.999).

## Discussion

The main aim of the current study was to investigate whether individual exercise intensity and the rate of O_2_ uptake could be accurately determined in upright and supine cycling and subsequently used to inform task-specific training prescriptions based on corresponding PO’s. To this end, all participants completed two moderate-intensity step-transitions from 20 to 100 W, followed by an incremental CPET and a verification bout (at 90% PPO) in both supine and upright positions. The main findings of the present study can be summarized as follows: (i) no differences were observed in the V̇O_2_ MRT between the two positions, although the τV̇O_2p_ was 7 s slower (*p* = 0.001) in supine compared to upright position; (ii) lower V̇O_2_ in the supine position at GET and RCP compared with the upright position; (iii) a ~6% lower V̇O_2_ peak (*p* = 0.002) was attained during supine cycling, but surprisingly comparable with the upright position (~3.6 L·min^−1^) during the constant work rate test when both conditions reached a V̇O_2_ peak corresponding to 90% PPO.

Determining exercise intensity domains for well-tolerated longer durations of exercise is challenging, both during the upright (Meyler, Bottoms & Muniz-Pumares, 2021; Keir, Pogliaghi & Murias, 2018) and supine cycling model. Compared to upright, supine exercise is characterized by slower O_2_ transport (Dillon et al., 2021), greater skeletal muscle deoxygenation (Goulding et al., 2021a), and a greater type II fibre activation cycling (Goulding et al., 2021a, 2021b), altogether likely leading to earlier exercise cessation. Contrary to our hypothesis, the V̇O_2_ MRT was not different between body positions likely due to an increase in the phase II-time constant (τV̇O_2p_) that was counteracted by a decrease in the circulatory time delay (TD of 3 s, Table 2) hindering the difference in V̇O_2_ MRT between body positions during moderate-intensity step-transitions (from 20 to 100 W). Of note, TD has no physiological equivalent (Rossiter, 2011), and subsequently confounds an accurate identification of potential metabolic zones for supine cycling, even though the phase II of the τV̇O_2p_ kinetics were significantly slower in supine moderate intensity step-transitions. Phase II τV̇O_2p_ kinetics play an essential role in exercise tolerance determination by mediating the PO at which muscle metabolite accumulation exceeds removal occurs–commonly defined as “ceiling” of tolerable endurance exercise (Goulding et al., 2021c), thereby preventing increased perception of effort and premature exercise cessation. A plausible explanation for the differences found (7 s, Table 2) would be that the supine cycling leads to kinetic disassociation between muscle O_2_ uptake and V̇O_2_ uptake, primarily caused by the lower baseline perfusion and sluggish O_2_ delivery kinetics during supine exercise (Rossiter, 2011). Moreover, besides the well-known methodological challenges (*e.g*., including low reproducibility, cardiorespiratory fitness status and pedal rate) in the MRT calculation due to the left-shift, the rate of O_2_ uptake and the role of the TD requires major consideration (Keir, Pogliaghi & Murias, 2019), especially when adjusting the V̇O_2_/PO from a single CPET supine session (Boone & Bourgois, 2017).

While different V̇O_2_ peaks between the two body-positions are plausible (Dillon et al., 2021), the scientific intrigue on whether the exercise intensity domains are different, remains. Here we sought to implement methodological procedures and to determine whether well-accepted markers of exercise intensity including the GET and RCP are transferrable from one position to the other, when corrected for V̇O_2_ MRT. Significantly lower V̇O_2_ uptake at GET and RCP were found for the supine compared to upright cycling (Table 3), and further discrepancy between V̇O_2_ uptake at GET and RCP in different body-positions can be observed in the Bland-Altman plots in Fig. 1 (panels C&D). Specifically, the differences in V̇O_2_ uptake between upright and supine postures at GET and RCP observed in our study (Table 3) are consistent with a reported ~10% higher V̇O_2_ at RCP in an upright condition (Goulding et al., 2021c). In parallel to V̇O_2_ uptake at RCP, Goulding et al. (2021c) measured the NIRS-derived deoxy[Hb] of the knee extensors to show that skeletal muscle deoxygenation profile and breath-by-breath O_2_ uptake, do not share the same underlying physiological mechanisms in different body postures. However, these findings are inconsistent with work by Keir et al. (2015) using a classical, upright cycling model to corroborate that RCP and NIRS-derived deoxy[Hb], among other exercise intensity indicators, occur at similar V̇O_2_ dependent on a common physiological phenomenon. The discrepancies between the studies can be explained by the loss of hydrostatic pressure and muscle perfusion during supine cycling, which reduces the pressure head for myoglobin-facilitated O_2_ diffusion, thereby limiting the rate of the O_2_ uptake increase at the onset of exercise (Poole, Musch & Colburn, 2022). An additional explanation implies that the V̇O_2_ determined at GET or RCP typically lags behind the true metabolic demands of that given intensity (*e.g*., PO) above the GET during classical upright cycling. At times, even if the V̇O_2_ MRT and the loss of mechanical efficacy between the GET and RCP are accounted for Keir et al. (2018) and Keir, Pogliaghi & Murias (2018). This typically leads to a situation where the ramp-identified PO elicits a metabolic intensity greater than the highest metabolic rate at which prolonged exercise is sustained, resulting in loss of locomotion efficiency, premature fatigue development and exercise cessation, which further exacerbate the determination of supine exercise intensities.

Recently, posture-related changes in O_2_ transport and V̇O_2_ peak were investigated during exhaustive CPET in both upright and supine positions (Dillon et al., 2021). Significantly lower peak cardiac outputs (computed from peak HR and peak stroke volume) were observed during supine cycling compared to upright and semi-supine postures *via* Doppler echocardiography. In agreement, we found a 9-beat lower HR max. on average during maximal CPET in supine compared to upright posture. Bringard et al. (2010) reported that following a 35-day bed rest, V̇O_2_ peak declined by 38% and 17% in the upright and supine postures, respectively. Interestingly, the V̇O_2_ peak decreases in the upright posture were predominantly explained *via* cardiovascular deconditioning (cardiac output ↓45%), whereas the cardiovascular responses during supine CPET remained unchanged. The authors postulated that impaired peripheral O_2_ utilization and diminished skeletal muscle oxidative capacity likely underpinned the substantial decline in the supine VO_2_ peak after 35-days of bed rest exposure (Bringard et al., 2010), while different mechanisms were responsible for the significant posture-related difference in V̇O_2_ decline during upright exercise. These claims have recently been confirmed with skeletal muscle bioenergetics during supine CPET by Goulding et al. (2021a, 2021b), who showed that the supine V̇O_2_ peak is partially limited *via* impaired O_2_ extraction fraction of the superficial knee extensor muscles, as observed from the greater rate of muscle deoxygenation at workloads above 165 W during supine cycling. It was suggested that O_2_ extraction is the limiting factor of oxygen uptake at higher supine intensities (especially associated with prolonged bed rest). Hence, there are misleading determinations of exercise intensity domains during supine cycling as a result of the lack of agreement around intensity domains determined during upright cycling.

Emerging evidence points to the notion that testing specificity is necessary during CPET procedures that inform exercise prescription and to avoid the methodological pitfalls of assuming exercise prescription is the same for different body-positions. Overall, considering the conflicting evidence observed between this study and the existing literature, further research is warranted in this area, since the commonly applied approaches may erroneously mislabel indicators of exercise intensity (including GET and RCP) that may otherwise have been correctly identified by the inclusion of the V̇O_2_ MRT. Individuals are thereby prescribed inappropriate exercise intensities. Moreover, the issue pertaining TD should not be overlooked when attempting to translate a given metabolic rate (*e.g*., the V̇O_2_ uptake) into a corresponding task specific PO, especially for the supine variant.

The current cross-sectional study is not exempt from limitations. A series of constant work-rate cycling bouts to determine critical power or maximal lactate steady state would have provided additional insight regarding position-specific markers of fatigue development. Although initially considered, such protocols were unfortunately discarded due to concerns of a high dropout rate because of Covid-19 infections or lockdowns. The practical application of a verification bout conducted at 90% PPO seems problematic and relates, potentially, to the methodological weakness of 90% PPO (see Niemeyer, Leithäuser & Beneke, 2020), but could be resolved by a verification bout performed at supramaximal PPO.

## Conclusions

In conclusion, significant differences in the V̇O_2_ uptake at GET, RCP and V̇O_2_ peak between body positions were observed in healthy young men. Yet, the V̇O_2_ MRT was not different between body positions, although the τV̇O_2_ kinetics lagged behind the actual metabolic demands, which should be taken into account when attempting to prescribe individualized exercise. Overall, our work adds to the on-going debate on exercise prescription by indicating that the inclusion of moderate-intensity step-transitions prior to a maximal CPET procedure is a viable and appropriate addition to ensure accurate determination of exercise-intensity domain(s). Further research is warranted to target the exact metabolic rates at which exercise is well tolerated for longer durations and to explore different methodological pitfalls associated with the exercise intensity determination at GET and RCP in supine posture, as well as the physiological mechanisms governing central and peripheral factors that limit O_2_ transport in different populations of varying cardiorespiratory fitness level.

## Supplemental Information

10.7717/peerj.13199/supp-1 Supplemental Information 1 Schematic illustration of the data collection protocol during upright and supine cycling.

10.7717/peerj.13199/supp-2 Supplemental Information 2 A representative trace of the pulmonary oxygen uptake kinetics. Panels A & B, upright *vs* supine body positions during on-transient cycling exercise

10.7717/peerj.13199/supp-3 Supplemental Information 3 Raw data.

## Additional Information and Declarations

### Data Availability

The following information was supplied regarding data availability: The raw data are available in the Supplemental File.
