# Supplementary figures and images for: Determination of exercise intensity domains during upright versus supine cycling: a methodological study
Source: PeerJ. 2022 Apr 13;10:e13199. doi: 10.7717/peerj.13199 (PMC9013233; doi:10.7717/peerj.13199)

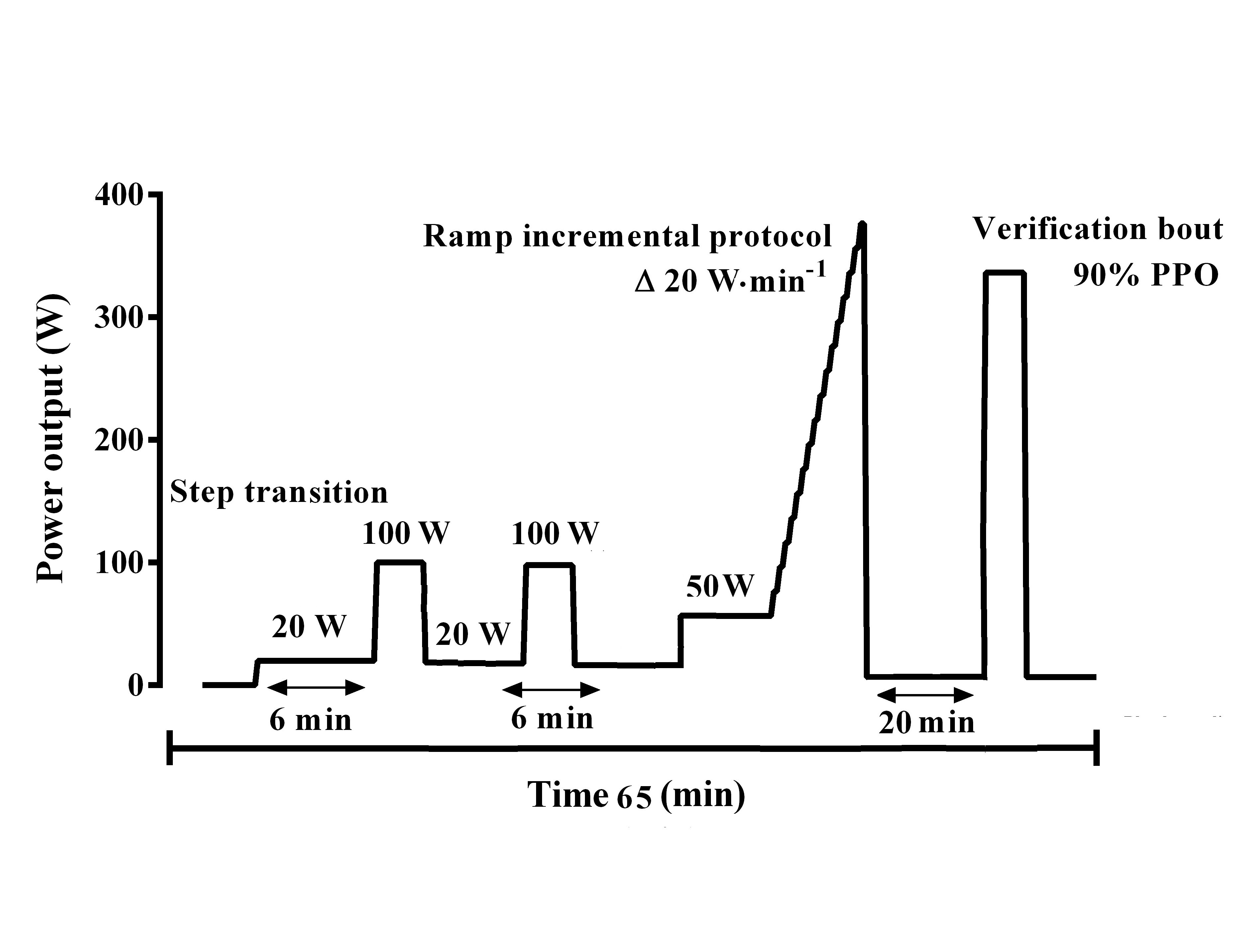

Supplement: Supplemental Information 1 [file peerj-10-13199-s001.jpg]

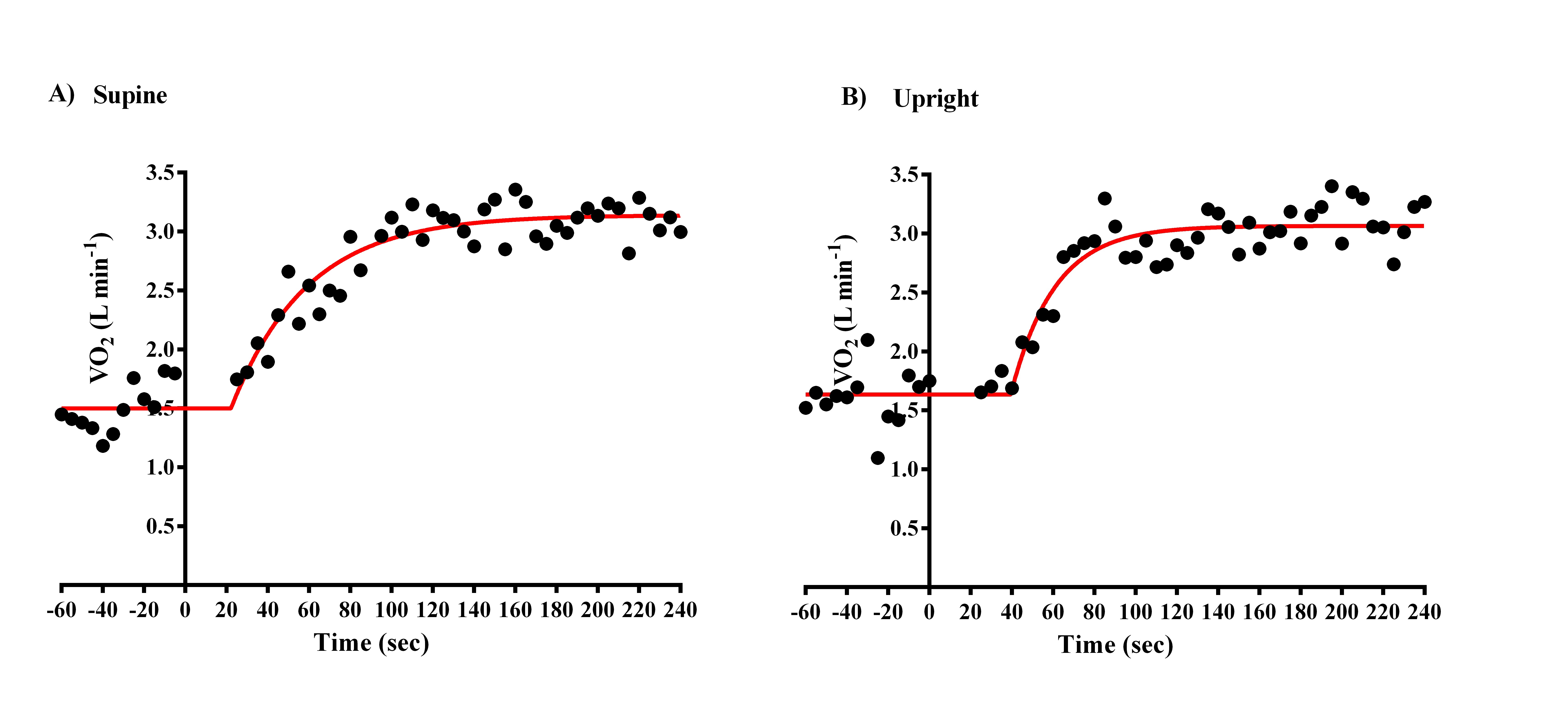

Supplement: Supplemental Information 2 — Panels A & B, upright vs supine body positions during on-transient cycling exercise [file peerj-10-13199-s002.jpg]
